# Cytotoxic and biological effects of bulk fill composites on rat cortical neuron cells

**DOI:** 10.1007/s10266-018-0354-5

**Published:** 2018-03-28

**Authors:** Hakan Kamalak, Aliye Kamalak, Ali Taghizadehghalehjoughi, Ahmet Hacımüftüoğlu, Kemal Alp Nalcı

**Affiliations:** 1 Department of Restorative Dentistry, Faculty of Dentistry, Fırat University, Elazig, Turkey; 2 Department of Endodontics, Faculty of Dentistry, Fırat University, Elazig, Turkey; 3 Department of Medical Genetic, Faculty of Medicine, Atatürk University, Erzurum, Turkey; 4 Department of Medical Pharmacology, Atatürk University, Erzurum, Turkey

**Keywords:** Apoptosis, Cell viability, Cytotoxicity, Total antioxidant capacity, Total oxidant status

## Abstract

The aim of this study was to investigate potential cellular responses and biological effects of new generation dental composites on cortical neuron cells in two different exposure times. The study group included five different bulk-fill flow able composites; Surefil SDR Flow, X-tra Base Flow, Venus Bulk Flow, Filtek Bulk Flow and Tetric-Evo Flow. They were filled in Teflon molds (Height: 4 mm, Width: 6 mm) and irradiated for 20 s. Cortical neuron cells were inoculated into 24-well plates. After 80% of the wells were coated, the 3 µm membrane was inserted and dental filling materials were added. The experiment was continued for 24 and 72 h. Cell viability measured by MTT assay test, total antioxidant and total oxidant status were examined using real assay diagnostic kits. The patterns of cell death (apoptosis) were analyzed using annexin V-FITC staining with flow cytometry. Β-defensins were quantitatively assessed by RT-PCR. IL-6, IL-8 and IL-10 cytokines were measured from the supernatants. All composites significantly affected analyses parameters during the exposure durations. Our data provide evidence that all dental materials tested are cytotoxic in acute phase and these effects are induced cellular death after different exposure periods. Significant cytotoxicity was detected in TE, XB, SS, FBF and VBF groups at 24 and 72 h, respectively.

## Introduction

Restorative materials with different characteristics are produced by today’s advanced technology in dentistry. It is preferred for these materials to have biocompatible on oral tissues [1–5]. While assessing the biological effects of restorative materials, the detrimental effects of these materials should be identified [6].

The composites used in dentistry consist of different conventional methacrylates such as bisphenol A-glycidyl methacrylate (Bis-GMA), triethylene glycol dimethacrylate (TEGDMA), urethane dimethacrylate (UDMA) and hydroxyethyl methacrylate (HEMA). These monomers are the major organic structures of the composite resins [7–12].

The clinical success of dental materials strongly based on their biological compatibility [13]. The cytotoxicity of dental composite materials has been ascribed to residual monomers [14]. These released monomers are correlated with the soft tissues in the oral cavity and the dentin-pulp system in the cavity [7–12].

Recently, in vitro and in vivo studies showed that monomers have been interrelated with genotoxicity [14–19], estrogenicity [14, 17, 19, 20], immune system [19, 21, 22], hypersensitivity, cytotoxicity [14, 15, 23, 24] and the production of reactive oxygen species [23, 25]. Based on these literature data, it is necessary to know biological and chemical effects of dental materials on cells. For this purpose nine different biological parameters were analyzed in this study in detail.

Human teeth have a large neurosensory system that is predominantly provided by the sensorial nerve fibers of trigeminal ganglion. Sensory nerve fibers progress from the tooth apex towards the enamel and innervates the surrounding tooth pulp [26–28]. Dense nerve fiber network in the pulp-dentin interface generate the raschkow subodontoblastic plexus and these nerve endings are provided to progress radially throughout the odontoblastic layer [29]. These sensorial nerves mediate nociception and forms the basis of a toothache. Problems in neuron cells occur and permanently damage the cell. The total number of neuron cells in the pulp is very important for tooth protection and immune defense [30].

Cytotoxicity in the restorative materials causes the formation of cellular stress [31, 32]. The formation of cellular stress leads to the observation of free radicals, formation of apoptosis and tissue destructions [31, 33–39]. The presence of Bcl-2 in the neuronal cells indicates that these proteins appear to inhibit apoptosis. The proteins of the Bcl-2 heterodimerize and homodimerize with each other, and the relative proportions of these gene families define whether or not a cell becomes apoptotic [40]. When a cell goes through apoptosis, viability and functionality are not possible of a tooth [41]. Thus, in the present study the effect of restorative materials on Bcl-2 gene expression on neuron cells was assessed.

Human beta defensins (HBDs) suppress proinflammatory cytokines. They activate the mast cells and perform degranulation. HBDs regulate the complement system, glucocorticoid production and antigen-specific immune response [42]. Four HBDs are isolated (HBD-1,-2,-3, and -4) until now. Although HBDs are observed in various tissues and organs, these peptides are mostly originated from epithelial cells [43, 44]. It is shown that epithelial cell-like odontoblastic cells structurally located inside the dentinal tubules of the teeth. These odontoblastic cells contain HBD-1 and HBD-2. Stimulation of odontoblast cells and microbial lipopolysaccharides with recombinant HBD-2 causes a decrease of HBD-1 and an increase of proinflammatory cytokines; IL-6 and IL-8. In addition, HBD-2 stimulates odontoblastic differentiation by increasing the dentin sialo-phosphoprotein expression [45, 46].

The studies on the cytotoxic effects of restorative materials were found in the literature review, but there was no comprehensive study assessing the biological effects of the materials. When cytotoxic effects were analyzed in the scientific studies, generally one or two parameters were investigated, but it is not enough to evaluate one or two parameters to determine the toxicity of a material. For this purpose biocompatibility tests should be supported by several parameters so that the authenticity of the research and correctness of the results increase.

In this study, we investigated potential cytotoxic and biological effects of bulk fill composites on neuron cells. We tested the viability of cells exposed to dental bulk fill composites that were widely used in dental clinics. Further, different biological parameters; total antioxidant capacity, total oxidant status, apoptosis level, IL-6, IL-8 and IL-10 cytokines, β-defensins: HBD-1 and HBD-2 were evaluated.

## Materials and methods

### Specimen preparation

After placing the restorative materials inside the Teflon molds (4 × 6 mm), strip bands were attached on the lower and upper surfaces of the molds and they were pressed with the glass slide to get a smooth surface. Then, sample were polymerized for 20/40 s via LED light device (Elipar Freelight II; 3 M-ESPE, St.Paul, MN, USA) according to the instructions of the producing company. After the samples were hardened, their edges and surfaces were polished using the polishing discs (Soft-Lex; 3 M ESPE, St. Paul, MN, USA). Before the materials were placed in 24-well plates; they were sterilized under UV light for 2 h. This process was repeated for all the materials used in this study. The materials used in this study are shown in Table 1.

**Table 1:** Materials used in this study

	Abbreviation	Material name	Manufacturer	Material type	Matrix type	Filler content	Filler content %
Group 1	SS	Surefil SDR flow	Dentsply Caulk/ABD	Bulk fill flowable composite	Dimethacrylate Resin, BPADMA TEGDMA, UDMA BHT	Silicate glass, Silicate oxide Hybrid glass fiber	80
Group 2	XB	X-tra Base Flowable	Voco Cuxhaven GERMANY	Bulk fill flowable composite	BisGMA, BISEMA, UDMA and Procrylat	Zirconia, Silica particles, iterium trifluoride,	75
Group 3	VBF	Venus bulk flow	Voco Cuxhaven GERMANY	Bulk fill flowable composite	UDMA, EPBADMA	Ba–Al-F Silicate glass, YbF_3_, and SiO_2_	65
Group 4	FBF	Filtek bulk flow	3 M/ESPE—USA	Bulk fill flowable composite	UDMA, BisGMA, BISEMA, ProAcrylate Resins TEGDMA	YBF3 fillers, zirconium silica particles	64.5
Group 5	TEF	Tetric-Evo Flow Bulk Fill	Ivoclar Vivadent Austria	Bulk fill flowable composite	Dimethacrylate Resin	Glass particles, Theprepolymer, Itanium trifluoride, Mixed oxides	76

### Preparation of cell culture

#### Preparation of neurons

**Fig. 1:**
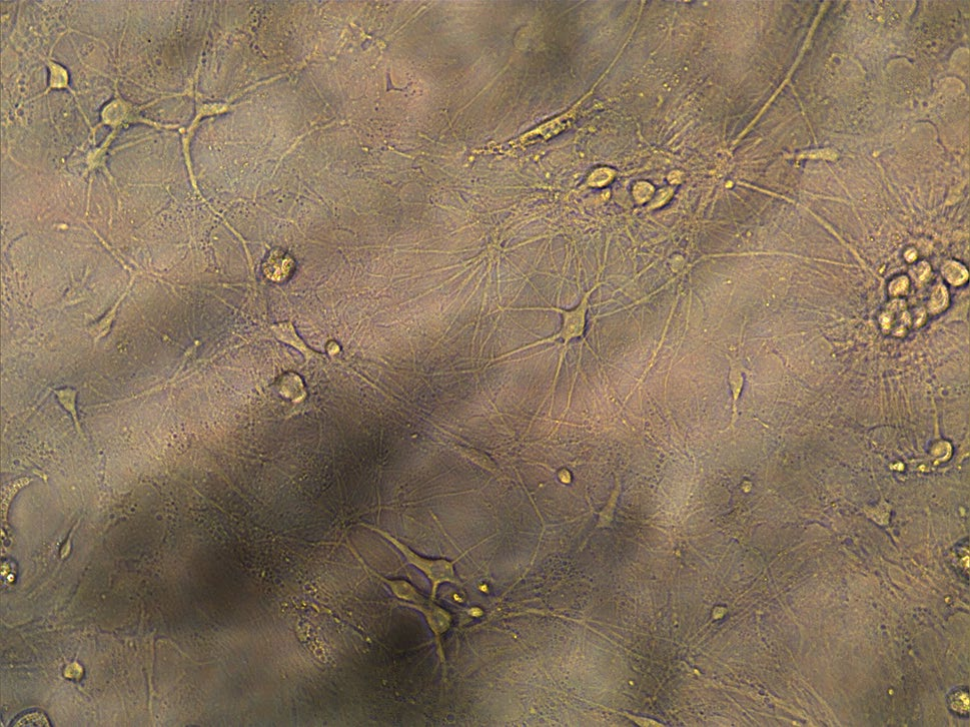
Cortex neuron cells with a 10 × magnification

Cortical Neuron cells (Fig. 1) were supplied from Atatürk University Department of Pharmacology. The cells coming in cryofalcons were dissolved in series at normal room temperature and centrifuged for 5 min in a 1200 rpm ^+^4 degrees centrifuge (Bachmann, Germany) by adding 2 cc NBM medium (Neurobasal Medium, Gibco, USA). The settled cells were mixed in the new medium (contains neurobasal medium, 1/10 FBS, 1/50 B27 and 0.1% antibiotic (penicillin–streptomycin-amphotericin B)), inoculated in 24-well plates and kept in the incubator containing 5% Co_2_ at 37°. The cells were included in the experiment when the dendrites completely covered the wells after 10 days [47].

### Preparation of insert membrane well plates

**Fig. 2:**
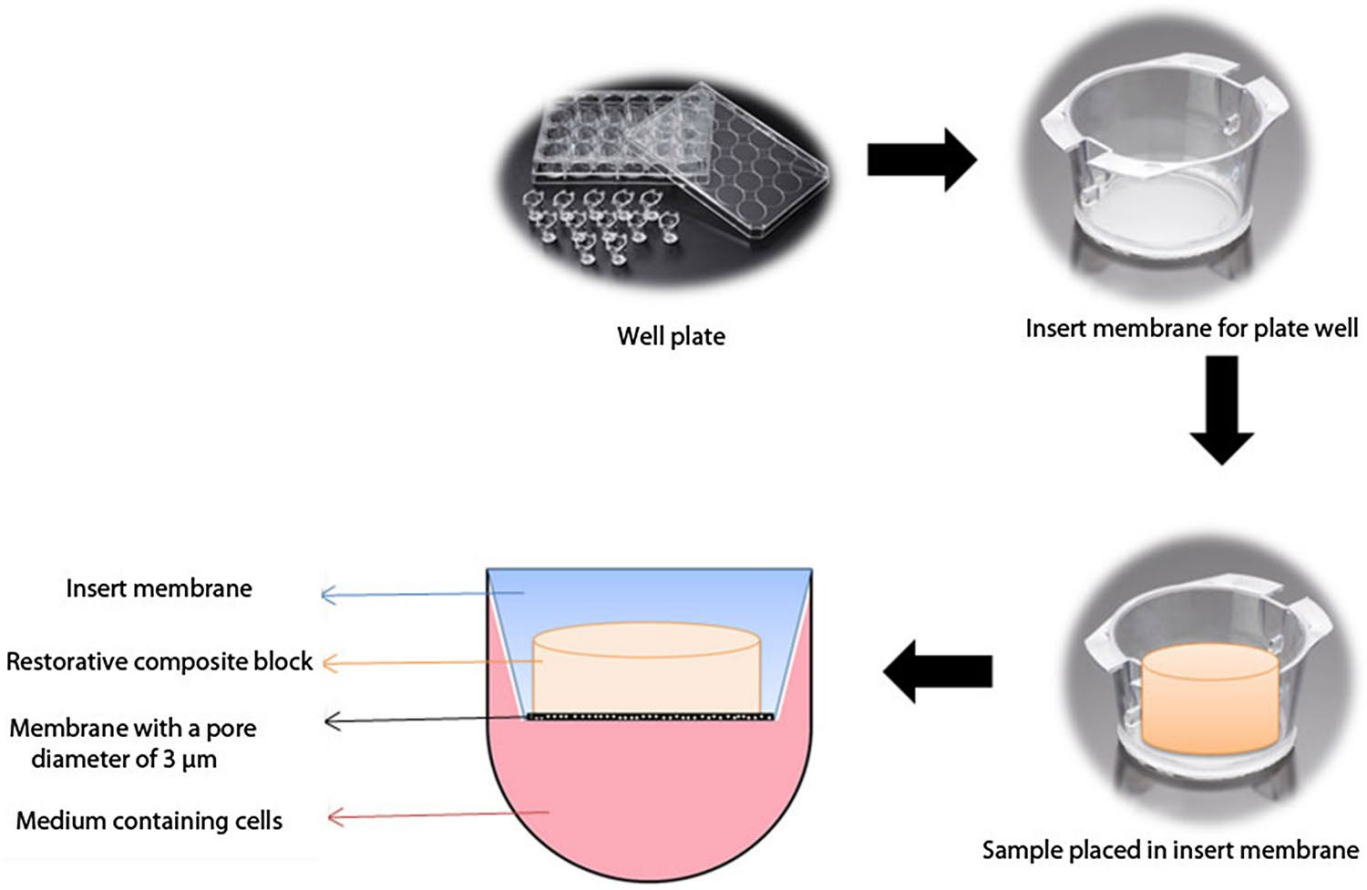
Mechanism of insert membrane system mimicking dentin tubules

Dentin tubule diameter is approximately 1 μm at the enamel-dentin border. Diameters of the dentinal tubules reach 3–4 μm on the pulp side [21]. Based on this information, insert membrane which have 3 µm pores were used in this study (Fig. 2).

The samples sterilized under UV light for 2 h were individually placed with the help of a sterile forceps to insert membranes in a sterile cabinet. Then, samples were placed into 24-well plates in which cells were located. The cells were left for incubation in the incubator of 5% CO_2_ at 37 °C during 24 h and 72 h (Fig. 2).

### Determination of BCl-2, HBD-1, HBD-2, IL-6, IL-8, and IL-10 expression

Samples were processed blindly for the detection of mRNA transcripts of HBD-1 and HBD-2. The total cellular RNA was isolated from each sample.

### RNA isolation

0.1 cc trypsin/EDTA was added to every medium of 24-well plates. After they were incubated in the incubator for 5 min, they were centrifuged at 1200 rpm during 5 min. The cells formed as a pellet and processed for RNA isolation and cDNA synthesis.

Isolation was performed using the qiagen RNA isolation kit. 1 cc Qiazole solution was added to the cells in the form of a pellet that were settled in the tube and they were kept at room temperature for 5 min. Then, 200 µl chloroform was added, agitated for 15 s, and kept at room temperature for 2–3 min. The samples were centrifuged for 15 min at the temperature of 4°. The colorless fluid at the top was transferred to another tube and it was vortexed by adding 1:1 ethanol alcohol. 700 µl of the sample was taken and placed in a collection tube and after it was closed, it was centrifuged at room temperature at 8.000*g* for 15 s. This stage was repeated once more. 700 µl RW1 solution was added into the RNeasy column. The cap of the column was closed and it was centrifuged at 8.000*g* for 15 s. Then, 500 µl RPE was centrifuged at RNeasy column at 8.000 g for 15 s. Then, 500 µl RPE was added to the RNeasy column and centrifuged at 8.000*g* for 2 min. After this stage, a new 1.5 cc tube was placed in the RNeasy column, 30–50 µl RNase free water was added and its cap was closed, it was centrifuged at 8.000*g* for 1 min.

### cDNA synthesis

For the cDNA synthesis; 2 µl from the genomic DNA wipeout buffer 7 × solution and RNA 1 µg and RNase free water were prepared to have a total volume of 14 µl and after they were kept at 42° for 2 min, they were again placed in the ice. Then, a total of 20 µl including reverse transcription master mix 1 µl, Quantiscript RT buffer 5 × 4 µl, RT primer mix 1 µl and RNA 14 µl were mixed and placed in the RT-PCR device. This was heated at 42° for 15 min and at 95° for 3 min and then it was kept up to – 20°.

### MTT, oxidant and antioxidant analyzes

#### MTT assay

Cell viability was analyzed by the MTT assay, which is based on the ability of the mitochondrial enzyme succinate dehydrogenase to convert the soluble tetrazo-lium salt (MTT) into formazan crystals in metabolically active cells. This water is stored in the cytoplasm of cells, and the color intensity is directly proportional to the amount of viable cells [48, 49].

To determine cell viability ratio of the materials, methylthiazolyldiphenyl-tetrazolium bromide (MTT) (sigma aldrich, USA) kit was applied at the end of 24 h and 72 incubation period. MTT solution (10%) was added to each well and incubated in the incubator containing 5% CO_2_ at 37 °C for 4 h. After 4 h medium plates removed and 100 µl DMSO (Dimethylsulfoxide) (sigma, USA) were added. The absorbance value was read at 550 nm wavelength in (optical density) a spectrophotometer device (μQuant, Bad Friedrichshall, Biotek).

$${\text{Viability}}\,\% \,{\text{ratio}} = \frac{{{\text{Sample}}\, {\text{absorbance}}\, {\text{value}}}}{{{\text{Control}}\, {\text{group }}\,{\text{absorbance}}\,{\text{value}}}} \times 100$$

### Total oxidant status (TOS)

In total oxidant status (TOS) assay, the assessment is done by measuring spectrophotometrically the density of the color related to the amount of oxidants in the sample. In the present study, TOS (Total Oxidant Status) kits manufactured by Rel Assay Diagnostics^®^ company (Turkey) were used.

The components in the kit were reactive 1 solution, reactive 2 solution, standard 1 solution, and standard 2 solution. To determine the TOS level; 500 µl Reactive 1 solution was added to the wells in which 75 µl plasma sample was present and after reading the initial absorbance value at 530 nm, 25 µl reactive 2 solution was added in the same well and second absorbance was read at 530 nm at the end of the waiting period of 10 min at room temperature. Then, we used the following formula to determine the TOS levels (mmol Trolox Equiv./L).

$${\text{TOS}} = \frac{{\Delta {\text{example}}}}{{\Delta {\text{ST}}2}} \times 20$$

ΔST2 (Δstandard 2 = ST2 s reading − ST2 first reading), Δ Sample (ΔSample = Sample second reading − Sample first reading).

### Principle of the total antioxidant status (TAS) measurement

In TAS assay; antioxidant capacity was determined by inhibiting formation of the 2-2′-azinobis (3-ethylbenzothiazoline 6-sulfonate = ABTS +) radical cation. In the assay process, real assay diagnostics^®^ (Turkey) commercial kit was used.

The components of the kit were reactive 1 solution, reactive 2 solution, standard 1 solution, and standard 2 solution. To determine the TAS level; 500 µl reactive 1 solution was added in the wells containing 30 µl sample and first absorbance was read at 660 nm. Then, 75 µl reactive 2 was added to the same wells and allowed to wait at room temperature for 10 min. At the end of the waiting period, second absorbance value was read at 660 nm. Then we used the following formula to determine the TAS levels (mmol Trolox Equiv./L).

$${\text{TAS}} = \frac{{(\Delta {\text{ST}}1 - \Delta \,{\text{example}})}}{{(\Delta {\text{ST}}1 - \Delta {\text{ST}}2)}}$$

ΔST1 (Δstandard 1 = ST1 s reading − ST1 first reading), ΔST2 (Δstandard 2 = ST2 s reading − ST2 first reading), ΔSample (ΔSample = Sample second reading- Sample first reading).

### Detection of apoptosis by Annexin-V binding

The AnnexinV-FITC apoptosis detection kit (Boivision) was used to identify connecting to Annexin V, which had a stable affinity for phosphatidylserine, in order to analyze apoptosis [50].

### Statistical analysis

IBM SPSS Statistics 22 (IBM SPSS, Turkey) program was used for the statistical analyses to assess the results obtained in the study. To analyze the data, OneWay ANOVA (OneWay Analysis of Variance) method was used and the significance values were compared with the control group. The significance values between the groups were assessed at the levels of *p* < 0.05 and *p* < 0.001 and a significant difference was found between the groups. We used power analyzed for stablieshed sample size (12 well for each sample).

## Results

### MTT analysis results after 24 h

**Fig. 3:**
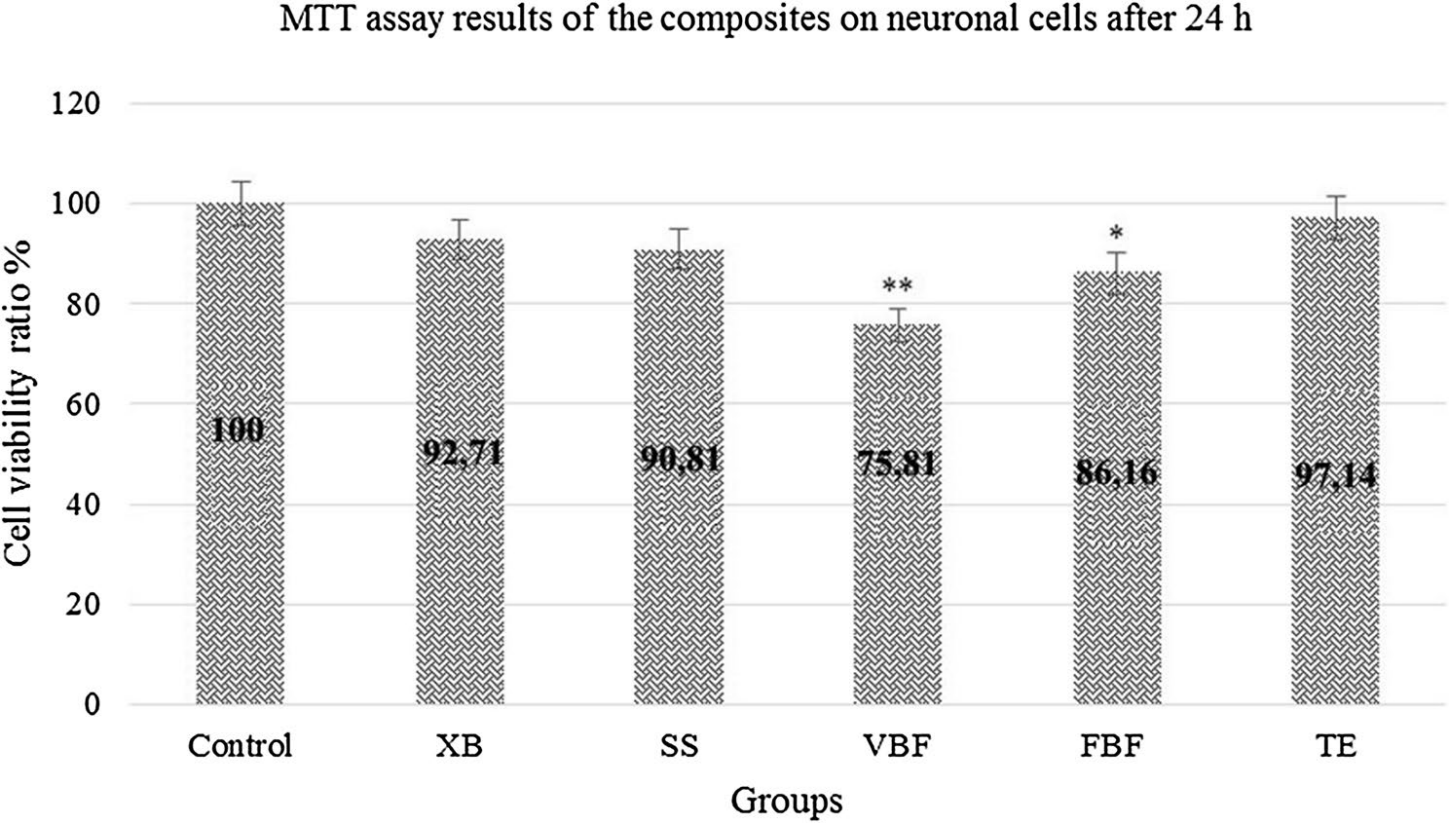
Viability rate of neuron cells exposed to different dental materials after 24 h. Each value was expressed as mean (*n* = 12). Results significantly differ from the mean of the control distribution at *p < 0.05 and **p < 0.001

When the XB and TE maintain cell survival within 24 h only compared to the control group; degeneration was observed in SS, FBF and VBF cells and the neurons in these groups got damaged after 24 h (Fig. 3).

### TAS and TOS analysis results after 24 h

**Fig. 4:**
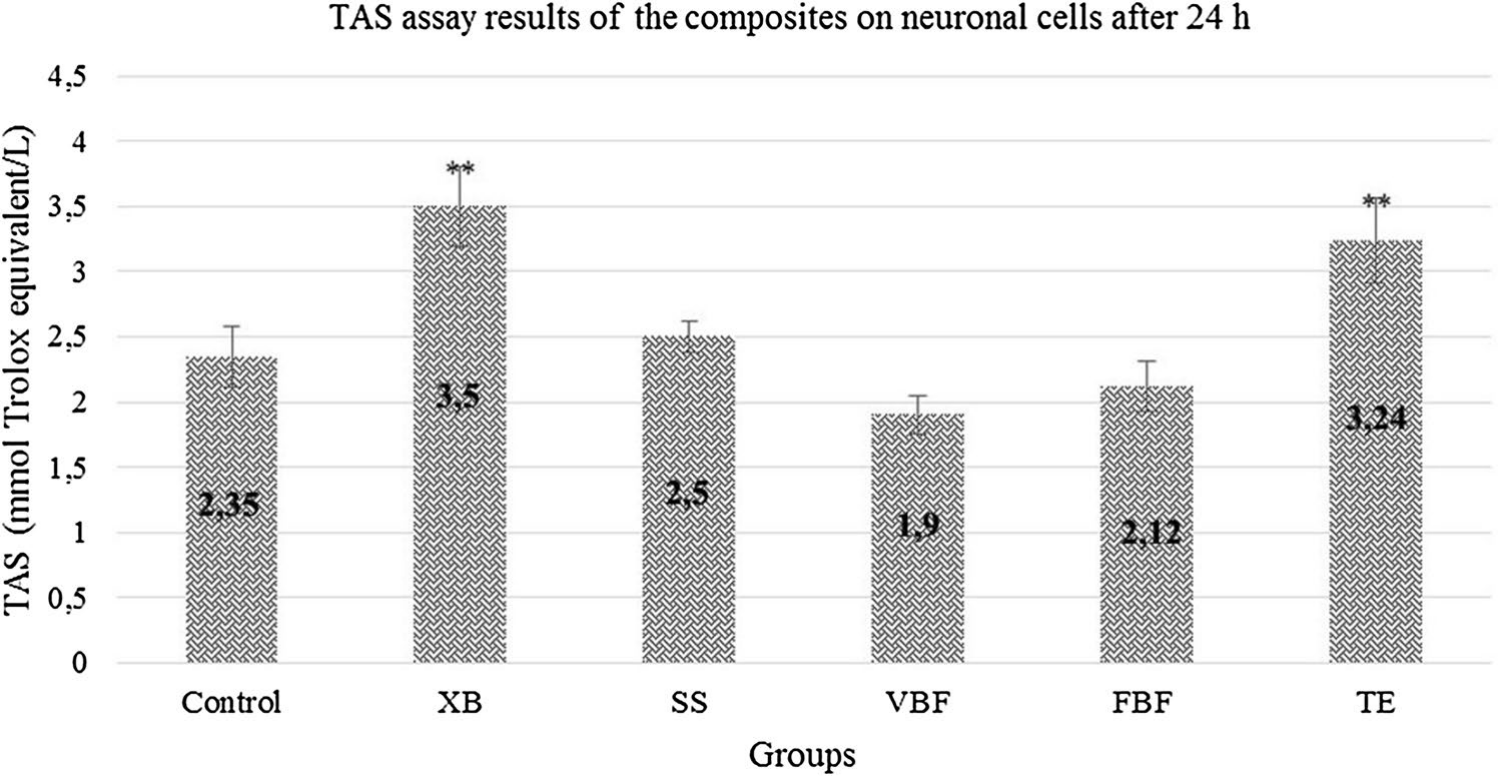
TAS levels of cells exposed to different dental materials after 24 h. Each value was expressed as mean (*n* = 12). Results significantly differ from the mean of the control distribution at *p < 0.05 and **p < 0.001

When the total antioxidant values were examined; a decrease was observed in the antioxidant status in the groups other than SS, XB and TE. TE, XB, and SS groups increased slightly more than the control group. In the other groups, antioxidant status decreased because the protection mechanism was deactivated in the cells. The cells in VBF group were more degenerated than the other groups (Fig. 4).

**Fig. 5:**
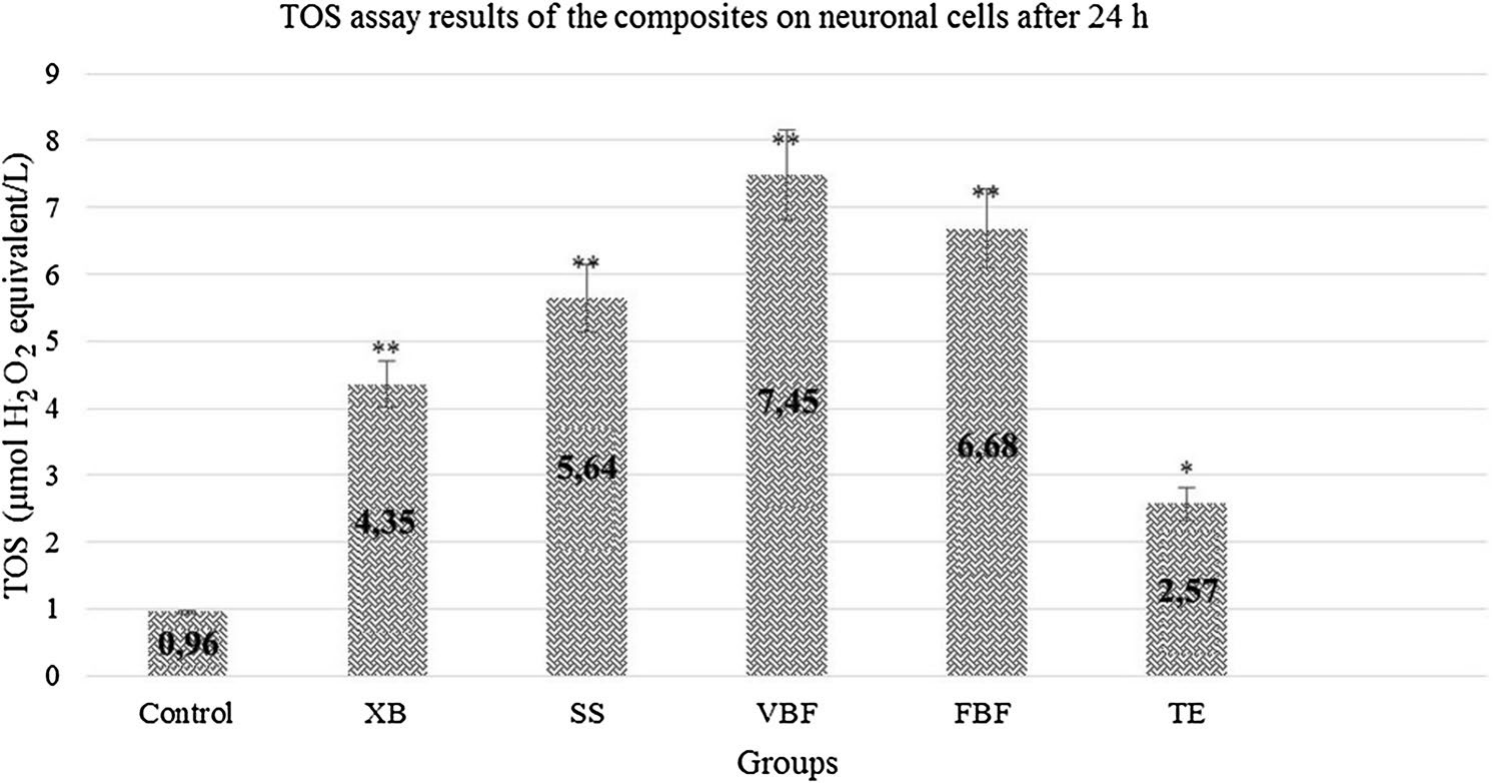
TOS levels of cells exposed to different dental materials after 24 h. Each value is expressed as mean (*n* = 12). Results significantly differ from the mean of the control distribution at *p < 0.05 and **p < 0.001

When the TOS values were examined; it was found that stress increased in the neurons in XB, SS, and TE groups but viability ratios were high (Fig. 5). Our data show accordance with the flow cytometry and cell viability data.

### Gene expression (IL-6, IL-8, IL-10, BCL-2, HBD-1, HBD-2, and ACTB) analysis results after 24 h

**Fig. 6:**
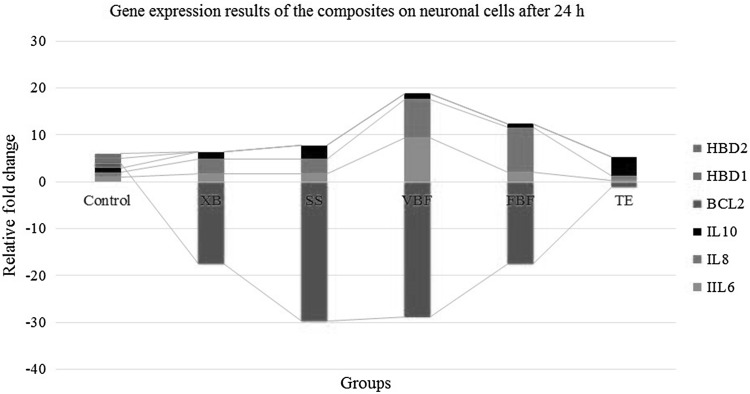
The comparison of gene expression results; Bcl-2, HBD-1, HBD-2, IL6, IL8, and IL10 with control group (housekeeping ACTB gene) after 24 h (*n* = 12)

While IL-6 values had an increase of 9 times in VBF group, they had an increase of 0.8-times in TE group. Similarly, IL-8 values were the highest in VBF group and the lowest in TE group. While defense cytokines (IL-10) increased in TE group, they decreased in both FBF and VBF groups. This negative decrease in BCL-2 value is an indication for their exposure to acute toxicity (Fig. 6). This decrease in BCL-2 value indicate an increase in apoptosis ratio.

### MTT analysis results after 72 h

**Fig. 7:**
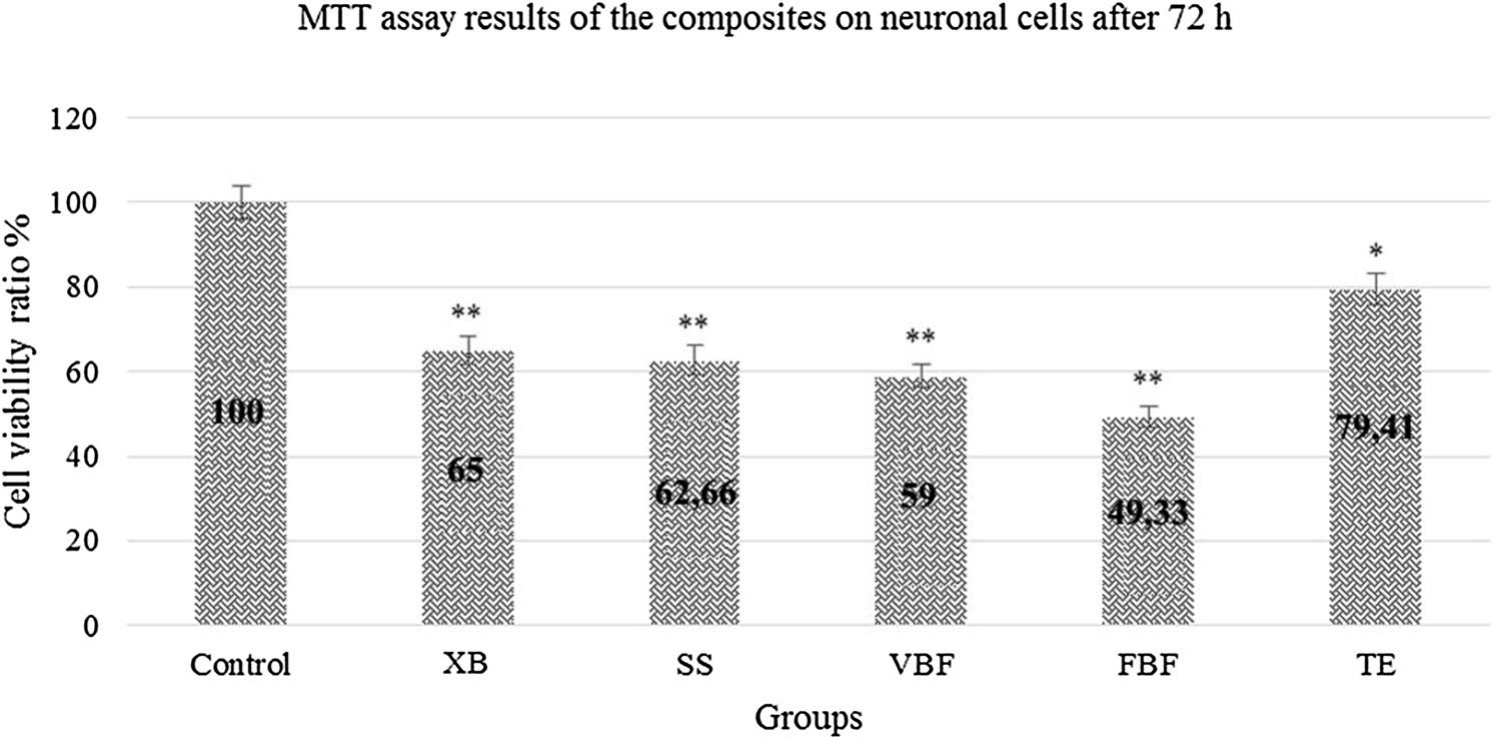
Viability rate of neuron cells exposed to different dental materials after 72 h. Each value was expressed as mean (*n* = 12). Results significantly differ from the mean of the control distribution at *p < 0.05 and **p < 0.001

Viability ratio of the cells was higher in TE when compared with the other groups. Within 72 h, XB could not tolerate the viability ratio and a decrease of 35% was observed in the viability ratio. Degeneration ratio in TE was 21%. 72 h later, TE material maintained the cell viability at the rate of 80% (Fig. 7).

### TAS analysis results after 72 h

**Fig. 8:**
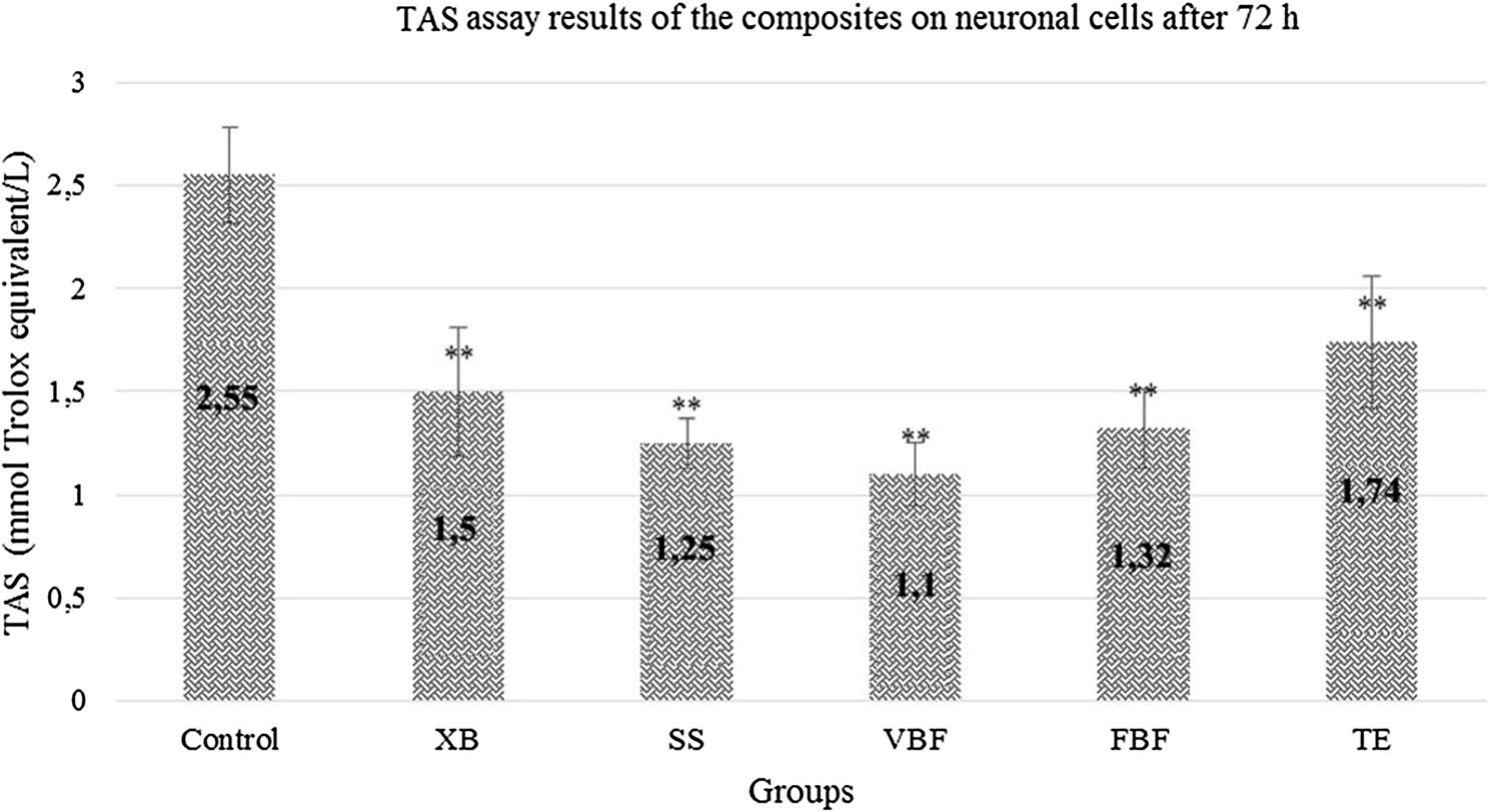
TAS levels of cells exposed to different dental materials after 72 h. Each value was expressed as mean (*n* = 12). Results significantly differ from the mean of the control distribution at *p < 0.05 and **p < 0.001

When the MTT results in the neurons were examined, viability values were low in VBF and FBF. The antioxidant values of VBF and FBF were very low when compared with the control group and cell degeneration was high. While the antioxidant value was 2.55 in the control group, it was 1.1 in VBF group and 1.32 in FBF group. This value was the lowest in VBF group (1.1) (Fig. 8).

### TOS analysis results after 72 h

**Fig. 9:**
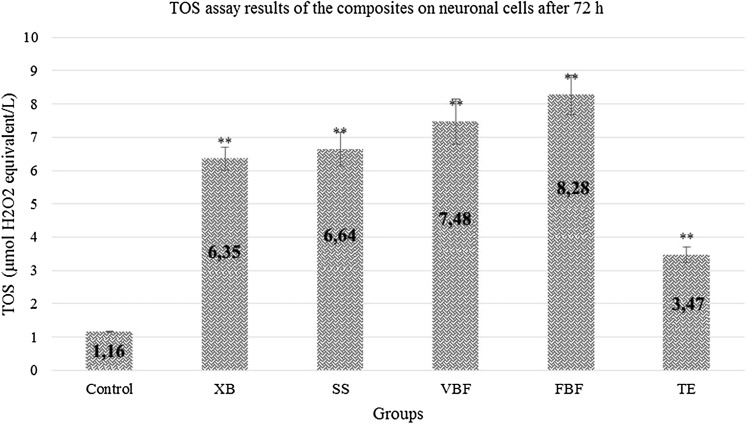
TOS levels of cells exposed to different dental materials after 72 h. Each value is expressed as mean (*n* = 12). Results significantly differ from the mean of the control distribution at *p < 0.05 and **p < 0.001

When the control group was examined in terms of neurons, TOS value was found to be 1.16 and a significant increase was observed in the TOS values in other groups. Oxidant levels of the groups other than TE increased approximately 3 times than the control group. Antioxidant values increased approximately 3 times in VBF, 3 times in FBF, and 3 times in XB when compared with the control group. In other words, this 3-times increase showed that there was an excessive stress in the cells. In the present study, no cellular degeneration was observed in XB and SS groups. They were not exposed to any stress. Other than XB and SS materials; XD, FBF and VBF showed that there would be an increase in the apoptosis ratio (Fig. 9).

### Gene expression (IL-6, IL-8, IL-10, BCL-2, hBD-1, hBD-2, and ACTB) analysis results after 72 h

**Fig. 10:**
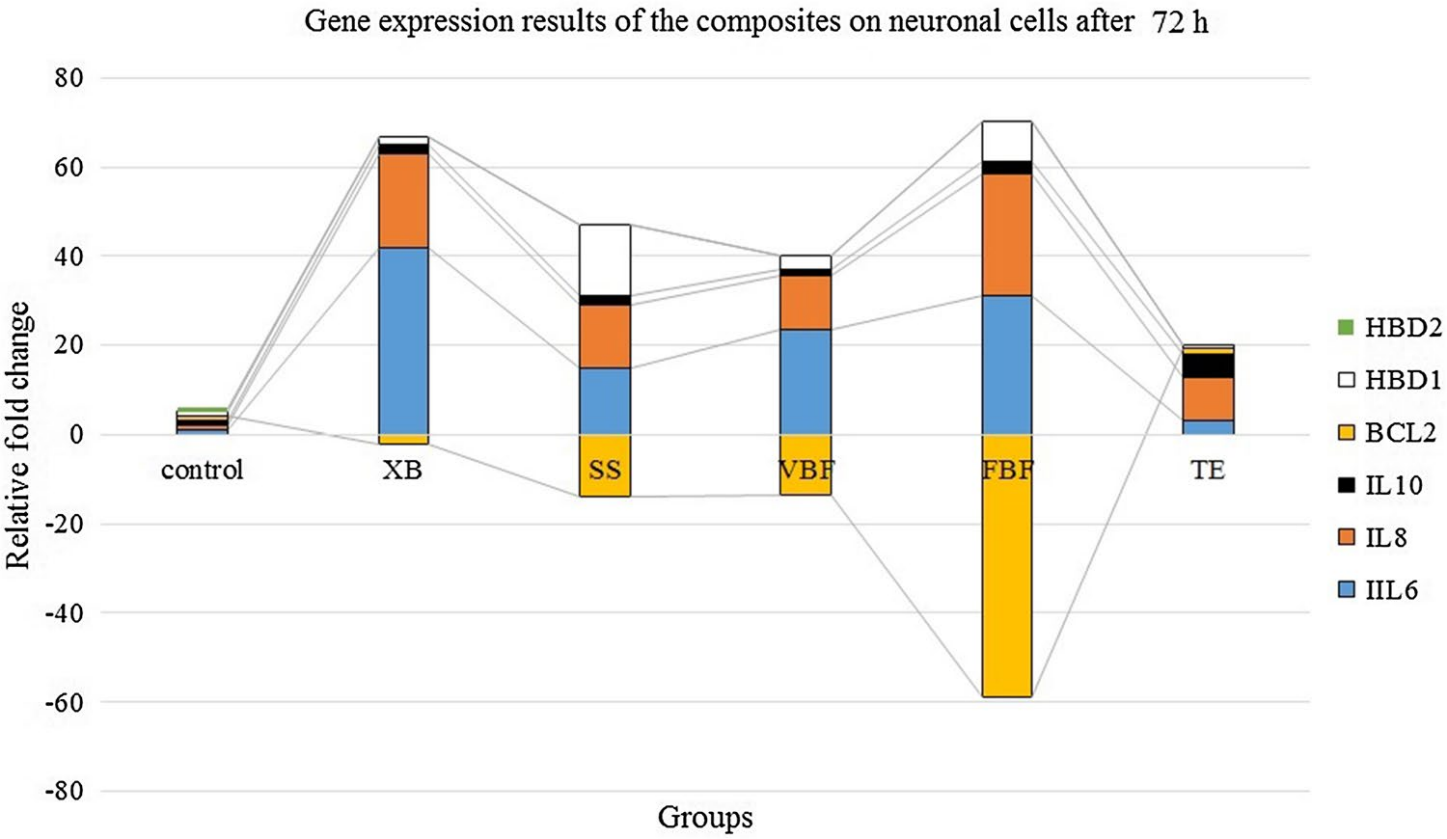
The comparison of gene expression results; Bcl-2, HBD-1, HBD-2, IL6, IL8, and IL10 with control group (housekeeping ACTB gene) after 72 h (*n* = 12)

When the genetic results of the neurons were assessed; it was found that while ACTB, HBD-1, IL-6, IL-10, BCL-2, and IL-8 genes were expressed, HBD-2 gene expressions cannot be taken. At the end of 72 h, there was an increase of 10% in almost all the genes. While IL-6 was 0.38 in TE at the end of 24 h, this value was 3.21 at the end of 72 h. Similarly, when the analysis results were assessed; while the IL-6 value in FBF was 1.98 after 24 h, it increased to 31.03 at the end of 72 h. When the analysis results were assessed; IL-8 value showed an increase at the end of 72 h. This showed that cellular breakdown was present. When the MTT results were examined, the increase in cellular degeneration over time verified the results of these parameters. When the IL-10 values were examined, no change was observed between 24 and 72 h. Because the IL-10 level is a protective mechanism, it remained constant but the increase in the inflammatory parameters signified that cell degeneration increased. While the cell ratio in TE was 97% at the end of 24 h, it was 79% after 72 h. Similarly, while the cell ratio in XB was 92% after 24 h, it decreased to 65% after 72 h. Since these results were compatible with each other, they verified the results of the study. When the BCL-2 values were examined, BCL-2 value was found to be positive only in TE group. In other groups, negative results were obtained and the genes were suppressed except for TE group. While BCL-2 value was the highest in FBF group (− 58, 79), it was the lowest in XB group (− 2.01). This parameter showed that rate of cellular death was the highest in FBF group and XB group had the lowest rate of cellular death among the other suppressed groups. When examining MTT results to verify the result, cell viability ratio at the end of 72 h was the lowest in XB group (49%). In other words, it was the group with highest cell degeneration. When the HBD-1 gene was examined, it was found that while there was an increase of 16% in SS, no increase was observed in TE group. While the cells in TE group maintained their viability, substantial cell degeneration occurred in other groups (Fig. 10).

## Discussion

During last decade, the use of bulk fill composite resins which consist of monomers and additives like photoinitiators, coinitiators, photostabilizers, inhibitors and inorganic fillers has tremendously been rising [51]. It is well known that the monomers may produce cytotoxic and biological effects as a result of incomplete cure [52, 53]. These effects are needed to be known to enlarge the use of newly produced composite resins.

Dental restorative composites should protect the viability of the pulp and associated cells. They should not show inflammatory reactions on the application site, and should not damage the tissue integrity [54].

Odontoblastic layer is the first step of the defense line. The odontoblast layer is also supported by a nociceptor network branching from the sensorial regions of the tooth pulp [27]. It is shown that bacterial pathogens activate the nociceptors forming one of the essential mechanisms causing neurogenic inflammation and pain [55]. As a response to harmful stimuli; the sensorial nerves in the tooth pulp are exposed to neuroplastic changes including the germination of terminal branches [27, 56]. In the cases that dentinal tubule infection is still limited, nociceptor germination shows itself by early nerve reaction against dentin damage [27]. In fact, the cross transition between the nervous system and the immune system is required for an effective defense against invasive situations [56]. It is shown that the sensorial denervation of the molar teeth of rats reduces the recovery ratio in dental pulp tissue and triggers a significant decrease in the chemotaxis of the immunity cells after the dentin damage [57]. Early neuroimmune response should be taken into consideration for the development of new approaches in dental treatments., otherwise, the deterioration of the condition will cause degeneration in the nerve cells and the accumulation of myelin debris in the pulp is inevitable in such cases. Peripheral nerve damage generally results in progressive infiltration of the immunocompetent cells, as the myelin debris accumulation is an obstacle in the recovery of axons and also is a major contributor to the inflammatory response after peripheral nerve damage [58]. Especially the myelin debris leads the macrophages and leukocytes mediating the clearance of tissue residues not to reach that site [59, 60].

Inadequate polymerization of the dental composite resins and resin-based bonding agents cause the free resin monomers stay in the resin matrix of the materials. These released resin monomers are correlated with the soft tissues in the oral cavity and the dentin pulp system in the cavity. In vivo and in vitro studies on the cytotoxic effect of residual monomers with associated tissues was proved by researchers [7–12, 61]. While assessing the biological effects of restorative materials, it is required to determine the harmful effects of the substances inside the material at first. This assessment can be done using the in vitro cytotoxic tests, genotoxic tests and gene suppression tests on the cell layers [62–64]. Cytotoxic effects on the neurons after the polymerization of new-developed composite materials can cause irreversible degenerations. Thus, the effects of the newly developed composites on the neuron lines in various periods of time were investigated by using different techniques in the present study.

There are studies using direct contact method to assess the cytotoxic effects of materials [65]. However, except for the MTT analysis, the insert membrane technique was used in the present study by simulating the dentinal tubules rather than direct contact of the cells with restorative materials. In this technique, membranes with pores of 3 micrometers are used and so, direct contact of the restorative materials with the cells is prevented. Thus, in the present in vitro study, a system that simulated dentin and allowed the diffusion of the test materials such as dentin was used, and thus the clinical status was imitated.

The cytotoxicity of a resin-based restorative material is deal with the amount of filler ratio and residual monomers disentangle from the materials. It is indicated that these monomers releasing from the resin matrix may cause biological and cytotoxic effects on dental cells over time [66, 67]. As a result of these literature research, the contents of the materials used in the present study were evaluated from cytotoxicity. When examining the filler ratio, it was observed that the least filler content was present in VBF and FBF groups. In this study, it was observed that VBF and FBF caused a more toxic effect in neurons and more degeneration when compared with the other materials. The decrease in the filler content made us think that it caused an increase in the material toxicity.

Recent work has indicated that in the groups, which have high molecular weight of ethoxylated bisphenol A dimethacrylate (BisEMA), is a matrix type of composite materials and more toxic effect was observed in these materials [68]. Similarly in the present study, the material contents of the groups were examined and it was observed that BisEMA was high especially in FBF and XB groups having high toxicity. Also similar to the results of the study assessing the effects of composites containing epoxy resin on inflammatory factors such as IL-6, IL-8, and TNF-α [69]; in this study the cytotoxic effects of flowable composites on neurons were investigated by assessing the cytokines, toxic effects were observed in epoxy resin groups and it was concluded that there was an increase in inflammatory factors.

It was reported that the contact period was another effective factor on the cytotoxicity of the materials. [19, 65]. But, in the present study, cortical neuron cells are used instead of rat pulp cells [50]. Previously, cytotoxic effects of the materials on human pulp stem cells via MTT analysis by using Venus bulk flow, Filtek supreme and Surefil SDR flow were assessed and as a result of this study the materials showed a slight cytotoxic effect [70].

In this study, SDR bulk-fill flow able composite showed a low toxicity in pulp neurons and cell viability ratio was found as 98% after 24 h. It was concluded that the materials caused a cellular degeneration of approximately 40% in the neurons after 72 h. In the present study, especially the toxic effect occurring within the first 24 h of the application of composite materials increased over time and a cell degeneration of 40% was observed in the other groups except for TE and XB groups [65, 71]. Glutamate toxicity is important in the degeneration of neurons. When the glutamate toxicity increases, positive ions start to penetrate into the cell. This degeneration can be tolerated when the negative ions penetrate into the cell as well as the entering positive ions. When TAS and TOS parameters were examined, it was observed that oxidant levels were tolerated because the high antioxidant status suppressed cell stress and cellular degeneration, and thus there was viability in the neurons.

IL-6 plays an important role in the defense mechanism and inflammatory processes [72]. IL-6 is secreted by cells, involved in host defense against infectious microorganisms and products, and damaged tissues [73]. IL-6 improves B cell differentiation and antibody secretion, T-cell proliferation, acute-phase protein synthesis, and the upregulation of phospholipase A2 [74]. Interleukin 6 also play a central role in gingival and periodontal inflammation [75]. It is desirable that the filler materials used in dental clinical trials should not induce IL-6 cytokine. In this study, an increase in IL-6 levels was observed in all groups after 72 h. After 72 h, IL-6 level was the least in the TE group whereas IL-6 level was the highest in the XB group. The percentage of fillers in both groups was found to be almost similar, so, it can be considered that matrix type is more effective than filler ratio of this difference. The level of IL-6 in the SS group (highest filler content) at the end of 72 h confirms that the increase in IL-6 level is more related to the type of matrix.

In this study, it is reported that Human β-defensins play an important role in the development of odontoblasts, which are responsible for formation of dentin, an agent responsible for local host defense and minor gene expression has been detected in HBD-2 in dental pulp cells in undecayed teeth and uninfected teeth [45].

Paris et al. investigated that while HBD-2 and HBD -3 were weakly expressed in healthy and inflammatory pulps, hBD -1 and hBD -4 significantly increased in inflammation compared to healthy pulps. They found that hBD-1 and hBD-4 played a role in pulpal host defense [76]. In this study; HBD-1 and HBD-2 were not expressed after 24 h in the neurons. After 72 h, the HBD-1 was expressed; whereas, HBD-2 was still not expressed. These expressions were not observed in the neurons because there were no HBD-1 and HBD-2 genes. In this study, we determined that degeneration was present in most of the cells over time. This was an indication for the fact that cellular degeneration was not caused by apoptosis, but by necrosis. The cells were directly degenerated before apoptosis. While assessing the cytotoxic effects of neuron cells, conducing the studies having an experiment period lasting for more than 72 h can provide more detailed information about the gene expression.

## Conclusion

This study provides new insights in the biological effects of composites on neuron cells in vitro. The results showed that the cytotoxic effects increased over time. However, it is not exactly known that which the changes occur in cells after 72 h. Although bulk fill composite resins are being improved, their cytotoxic effects became still got disadvantageous. Recently, composites without resin monomers have been applied to the dental market. Based on these concerns, considerable effort is being expended to develop alternative monomer systems which may have fewer negative biological consequences.

### Clinical relevance

Neuronal cells are responsible for the stimulation of reparative dentine and the pain sensation of teeth. Neuronal cells never grow proliferation, so the numbers of neuron cells are fixed. The reduction in neuronal cells shortens the long-term clinical course of the teeth. Therefore, it is important to evaluate the effects of composites on neuron cells instead of dental pulp and gingival fibroblast cells in cytotoxicity studies.
